# Dismissal of the utility of free cortisol measurement is premature: response to Cohen et al.

**DOI:** 10.1007/s00134-012-2507-2

**Published:** 2012-03-06

**Authors:** Nienke Molenaar, Albertus Beishuizen, Johan A. B. Groeneveld

**Affiliations:** Intensive Care, VU Medical Center, De Boelelaan 1117, 1081 HV Amsterdam, The Netherlands

Dear Editor,

The letter from Cohen and colleagues [1] referring to our recently published assessment [2] of adrenal (in)sufficiency of corticosteroid secretion raises the interesting debate of whether we should measure free cortisol levels in critically ill patients with a suspicion of critical illness-related corticosteroid insufficiency (CIRCI).

We did not estimate the free cortisol level with the help of the Coolens equation, which departs from normal albumin levels, and only roughly reflects true, measured free cortisol when carefully adapted for abnormal binding proteins in sepsis and shock, which, on top of this, may also be accompanied by changes in affinities [3]. Moreover, mathematical coupling may spuriously elevate correlation between free and total cortisol when free cortisol is calculated from total levels. We therefore directly measured free cortisol by an accepted reference standard technique (equilibrium dialysis) and still found a direct correlation with total cortisol, confirming the independent relation also observed by the authors of the letter (and other investigators as well). However, it was not this relation that led us to conclude that free levels hardly contribute to total levels in determining adrenal secretion. It was the cortisol response to 250 μg of ACTH that was somewhat larger for free than for total cortisol but nevertheless highly interrelated, relatively independently of binding proteins, that prompted us to conclude that delta total cortisol can substitute for delta free cortisol in assessing adrenal reserve, particularly in sepsis. Moreover, the differences in percentage increases in free and total cortisol were less when responses to ACTH were less and therefore did not interfere much with the diagnosis of CIRCI when using the delta total cortisol less than 250 nmol/L as a cutoff value. The concordance around the suggested cutoff of delta free cortisol of 76 nmol/L vs the delta total cortisol of 250 nmol/L defined by Cohen’s kappa was 0.83 (*p* < 0.001) in the septic and 0.58 (*p* < 0.001) in the non-septic patients, indicating almost complete interchangeability at least in sepsis. In fact, the ROC curve analysis we provided pointed to the excellent diagnostic (predictive) value of delta total for delta free cortisol upon administration of 250 μg of ACTH in sepsis.

Interestingly, the study by Cohen et al. [4] on only 29 septic patients failed to demonstrate a correlation between delta total and delta free cortisol, whereas we studied 63 septic patients. The discrepancy between these studies might be partially explained by Cohen et al.’s use of 1 μg ACTH. Also their baseline free cortisol levels were surprisingly low which may be related to the method they used (ultra-high-performance liquid chromatography–tandem mass spectrometry after ultrafiltration), introducing some error and explaining lack of correlation with delta total cortisol. These concerns also apply to their results in patients with liver cirrhosis where they also found a poor relation and predictive value of delta total versus delta free cortisol and the former seemed to overestimate (or the latter to underestimate?) adrenal insufficiency [5].

Finally, we demonstrated that a delta total cortisol less than 250 nmol/L was a better prognosticator of mortality than the delta free cortisol, and the superiority of the predictive value of delta free cortisol in the Cohen et al. study [4] was only evidenced after adjustments in multivariable analysis.

For these reasons we belief that our data collectively demonstrate that total cortisol increases upon administration of 250 μg of ACTH would suffice in assessing the adrenal (in)sufficiency of CIRCI, particularly in sepsis.
